# CLPP inhibition triggers apoptosis in human ovarian granulosa cells *via* COX5A abnormality–Mediated mitochondrial dysfunction

**DOI:** 10.3389/fgene.2023.1141167

**Published:** 2023-03-17

**Authors:** Xiong Yuan, Wenjie Ma, Shuping Chen, Huiyuan Wang, Chenyi Zhong, Li Gao, Yugui Cui, Danhua Pu, Rongrong Tan, Jie Wu

**Affiliations:** ^1^ Department of Obstetrics and Gynecology, The First Affiliated Hospital of Nanjing Medical University, Nanjing, China; ^2^ State Key Laboratory of Reproductive Medicine, School of Public Health, Nanjing Medical University, Nanjing, China

**Keywords:** CLPP, COX5A, granulosa cell (GC), apoptosis, POI

## Abstract

Premature ovarian insufficiency (POI) is characterized by early loss of ovarian function before the age of 40 years. It is confirmed to have a strong and indispensable genetic component. Caseinolytic mitochondrial matrix peptidase proteolytic subunit (CLPP) is a key inducer of mitochondrial protein quality control for the clearance of misfolded or damaged proteins, which is necessary to maintain mitochondrial function. Previous findings have shown that the variation in CLPP is closely related to the occurrence of POI, which is consistent with our findings. This study identified a novel CLPP missense variant (c.628G > A) in a woman with POI who presented with secondary amenorrhea, ovarian dysfunction, and primary infertility. The variant was located in exon 5 and resulted in a change from alanine to threonine (p.Ala210Thr). Importantly, Clpp was mainly localized in the cytoplasm of mouse ovarian granulosa cells and oocytes, and was relatively highly expressed in granulosa cells. Moreover, the overexpression of c.628G > A variant in human ovarian granulosa cells decreased the proliferative capacity. Functional experiments revealed that the inhibition of CLPP decreased the content and activity of oxidative respiratory chain complex IV by affecting the degradation of aggregated or misfolded COX5A, leading to the accumulation of reactive oxygen species and reduction of mitochondrial membrane potential, ultimately activating the intrinsic apoptotic pathways. The present study demonstrated that CLPP affected the apoptosis of granulosa cells, which might be one of the mechanisms by which CLPP aberrations led to the development of POI.

## 1 Introduction

Premature ovarian insufficiency (POI) is defined as the early cession of ovarian function in women before the age of 40 years; it is clinically characterized by menstrual disorders with high gonadotropin and low estrogen levels (Jiao et al., 2018). The incidence of POI is 1%–2.8% (Tiosano et al., 2019; Wu et al., 2014). POI significantly jeopardizes the physiological and psychological health of women (Chon et al., 2021). A clear etiological diagnosis is of great significance for patients and their family members, which is conducive to family planning.

The etiology of POI is complex and highly heterogeneous, including genetic, autoimmune, infectious, or medical factors. The abnormalities in chromosome number and structure account for 10%–13% of women with POI, and abnormalities in the number or structure of the X chromosome are the most common (Qin et al., 2015). In addition, monogenic identification has increased with the widespread use of next-generation sequencing, including mutations in various reproductive ligands and their cognate receptors (FSHR, BMP15, and GDF9), meiosis and DNA repair genes (MSH, MCM, and FANC), transcriptional factors (FIGLA, NOBOX, and FOXO), RNA metabolism and translation (FMR1 and CPEB1), and enzymes (POLG1 and CLPP) (Yang et al., 2021). Recessive CLPP mutations have been found to cause the human Perrault variant of ovarian failure and sensorineural hearing loss (Jenkinson et al., 2013), and global germline Clpp knockout female mice showed auditory deficits and complete infertility (Gispert et al., 2013).

CLPP is an abbreviation for caseinolytic mitochondrial matrix peptidase proteolytic subunit, which is located on chromosome 19p13 (Kang et al., 2005). Previous studies related to CLPP found that the infective or pathogenic capacity of *Salmonella typhi*, *Listeria monocytogenes*, and *Staphylococcus aureus* was significantly reduced in the absence of the ClpP protease system (Hensel et al., 1995; Mei et al., 1997). Recent studies have also found that the ClpP complex increases biofilm formation in *Porphyromonas gingivalis* by regulating the expression levels of fimA, mfa1, and luxS, thereby affecting the pathogenicity of bacteria (He et al., 2019). Interestingly, clpp deletion within the fungus improves the growth and development of the fungus and prolongs its lifespan (Fischer et al., 2013).

CLPP is highly conserved among species and is widely found in the mitochondria of eukaryotes. In mammals, it typically binds to AAA+ family molecular chaperones to form protease complexes. The members of the AAA+ family can use the energy provided by the hydrolysis of ATP to misfold protein substrates, which are then transferred to the hydrolytic cavity of the CLPP protease for degradation (Mabanglo et al., 2022; Ripstein et al., 2020). Mitochondria play an important role in oocyte energy homeostasis, and several mitochondrial enzymes have been identified in patients with POI, while CLPP is a known POI-causative gene. Defects in CLPP cause Perrault syndrome type 3 (PRLTS 3), with clinical manifestations of sensorineural deafness and POI (Faridi et al., 2022). The deficit in the reduction of mtDNA and mitochondrial numbers may underlie infertility in the CLPP variant of the recessive Perrault syndrome. A reduced number of granulosa cell layers and a higher number of apoptotic bodies were observed in *Clpp* ^−/−^ mouse follicles (Gispert et al., 2013). Furthermore, both cellular energy metabolism and mitochondrial dynamics seem to be severely affected in *Clpp* ^−/−^ mouse oocytes (Wang et al., 2018)**.** Folliculogenesis was not affected by the loss of Clpp in granulosa/cumulus cells (Esencan et al., 2020). However, the roles of CLPP in granulosa cells remain uncertain. The present experiments revealed the function and mechanism of CLPP in granulosa cells, which might provide more clues and references for exploring the pathogenesis of POI.

In the present study, we reported a consanguineous Chinese family in which one daughter was diagnosed with secondary amenorrhea and ovarian insufficiency with no sensorineural deafness features. Moreover, a novel missense variant at exon 5 of CLPP (c.628G > A, p. Ala210Thr) was identified by whole-exome sequencing. Further, we demonstrated at the cellular level that the inhibition of CLPP led to the accumulation of damaged or misfolded COX5A in ovarian granulosa cells, causing mitochondrial dysfunction and increased apoptosis.

## 2 Results

### 2.1 CLPP mutation was associated with POI in women

The proband was enrolled at the First Affiliated Hospital of Nanjing Medical University. She was 21 years old and had a brother in a Han Chinese family. Her menstrual cycles had become erratic with infrequent menses at the age of 18 years since menarche at 15 years of age. She had no clinical manifestations of hearing loss. Besides, no neurological symptom was present. The laboratory findings are shown in Table 1. Her basal hormone levels were abnormal, and the levels of anti–Müllerian hormone were also found to be lower. The analysis of blood test reports showed normal functions of thyroid, and the immunological investigations were in normal limits. The karyotype was 46, XX (Table 1). She was prescribed estrogen replacement therapy to promote uterine development and prevent osteoporosis and other complications. The patient was infertile; she eventually received egg donation at another reproductive medicine center and had a daughter.

**TABLE 1:** Clinical data of the proband.

Clinical information	The proband
Age (years) at molecular diagnosis	19
Somatic karyotype	46, XX
Sex hormones	
FSH (IU/L)	61.84
LH (IU/L)	30.1
Estradiol (pg/mL)	20.13
Testosterone (ng/dL)	15.11
Prolactin (ng/mL)	0.31
AMH (ng/mL)	0.01
Pelvic ultrasound	Small uterus and ovaries
Thyroid hormones	
TSH (mIU/L)	2.48
FT3 (pmol/L)	5.55
FT4 (pmol/L)	17.43
TPOAb (IU/mL)	11.01
TGAb (IU/mL)	<10
Information of CLPP mutations	
cDNA mutation	c.628G>A
Mutation type	Missense Mutations (0.999)
Protein alteration	p. Ala210Thr
Functional prediction	
SIFT	Damaging (0.012)
PolyPhen-2 (human div score)	Probably Damaging (0.972)
MutationTaster (probability of prediction)	disease causing (0.999)

**FIGURE 1:**
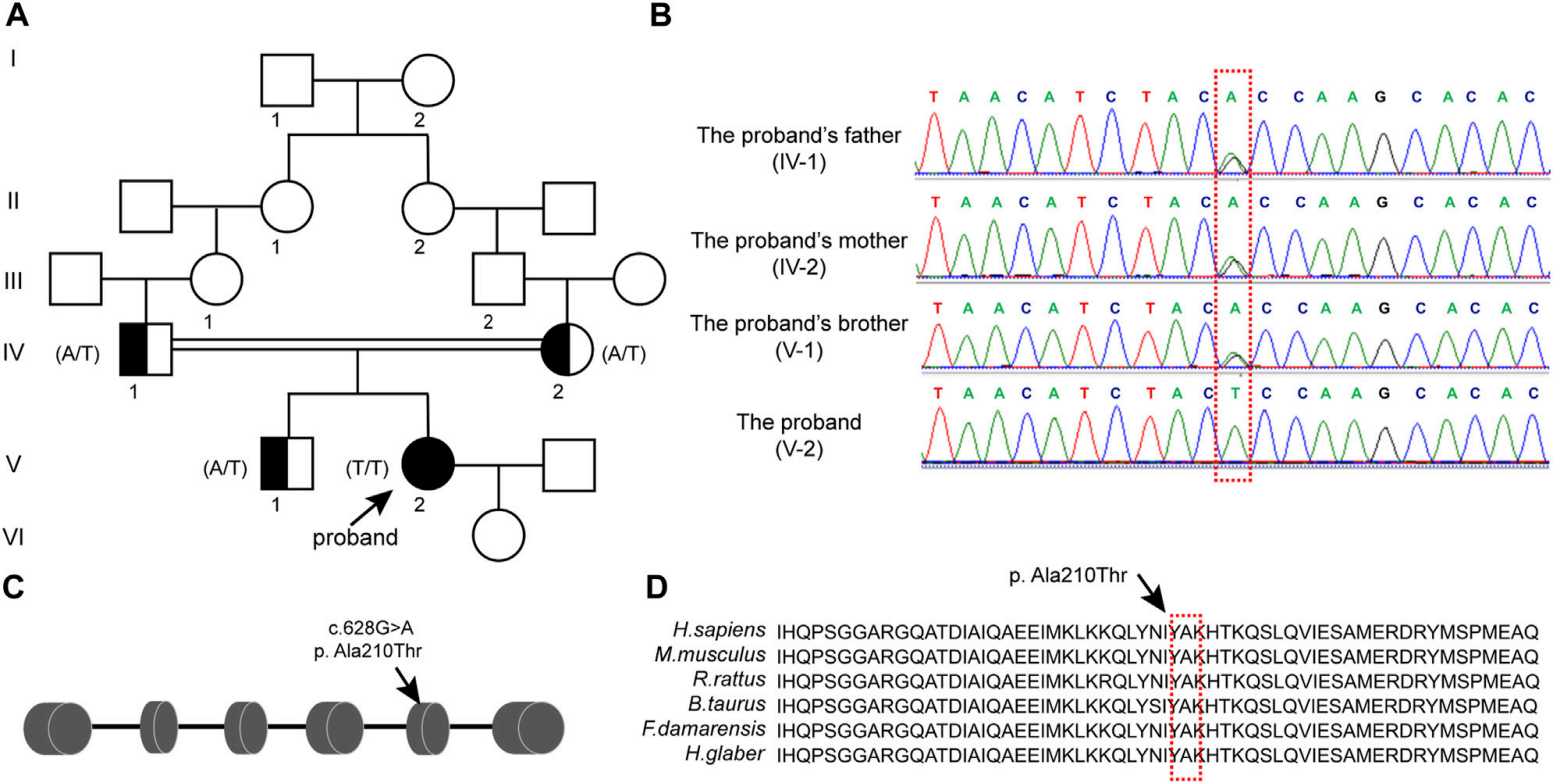
CLPP mutation was associated with premature ovarian insufficiency in women **(A)** Pedigree of a Han family with homozygous mutations in CLPP. Male family members are denoted with squares and female family members with circles. The arrow indicates the proband (V-2). Genotypes of the mutation c.628G>A are noted below the symbols. **(B)** Sanger sequencing verified the CLPP mutation, c.628G>A. The proband carried a homozygous missense mutation, and the red box shows the mutation location. Her parents and brother carried heterozygous alleles. **(C)** Schematic diagram of CLPP with the function domain and position of the identified mutation. CLPP gene contains six exons. The position of the missense mutation c.628G>A was in exon 5 of the CLPP gene, resulting in the replacement of the 210th amino acid alanine (Ala) by threonine (Thr), as indicated by the arrow. **(D)** Conservation of the mutation site in CLPP was studied across different species.

Their ancestral history was provided by the patient herself, and her parents had a consanguineous marriage. As shown in Figure 1A, the respective grandmothers of the proband’s parents were biological sisters. Her mother’s menses were basically normal, with no signs of ovarian dysfunction. Her brother was also infertile and presented with small testicular size, which was ultimately attributed to his chromosomal factors. His karyotype was 47, XXY.

We extracted the peripheral blood from this family for whole-exome sequencing and biological analysis to identify the potential mutation that might cause POI. The variant was a homozygous missense mutation c.628G > A (p.Ala210Thr) in exon 5 of the CLPP gene (NM_8192) (Figure 1C). Sanger sequencing was performed to verify the mutation (Figure 1B). The results demonstrated that the proband carried the CLPP homozygous mutation (V-2), and her parents and brother carried heterozygous alleles (IV-1, IV-2, and V-1), which was autosomal recessive (Figure 1A). Multiple sequence comparisons showed that the mutant loci of the CLPP ortholog were highly conserved across species (Figure 1D).

The functional pathogenic effects of the variants were predicted using the online bioinformatics tools SIFT, Polyphen-2, and MutationTaster. The predictions showed that the missense mutation at this locus of CLPP was probably damaging or disease-causing (Table 1).

### 2.2 Mutation of CLPP reduced the growth and viability of human ovarian granulosa cells

**FIGURE 2:**
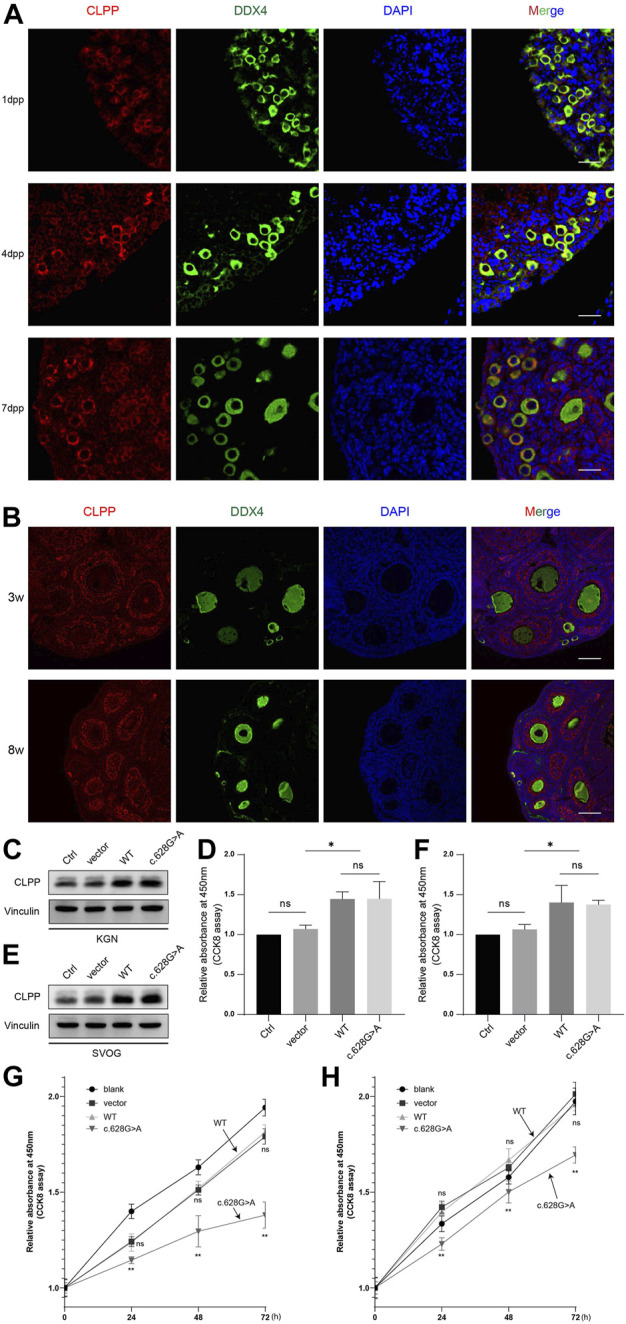
Mutation of CLPP reduced the growth and viability of human ovarian granulosa cells **(A)** Cellular localization of CLPP in newborn ovaries. Mouse ovaries were stained for CLPP (red) and the oocyte-specific marker DDX4 (green) at the indicated time points. Nuclei were counterstained with DAPI (blue). dpp is an abbreviation for days *postpartum*. **(B)** Cellular localization of CLPP in the ovaries of 3 W and 8 W mice. CLPP was localized mainly in the cytoplasm of oocytes and granulosa cells in growing follicles, with a relatively higher expression in granulosa cells. Scale bar, 100 µm. **(C,D)** Detection and quantification of the efficiency of transfection of wild-type CLPP(WT) and mutant CLPP (c.628G > A) in KGN cells. **(E,F)** Detection and quantification of the efficiency of transfection of wild-type CLPP (WT) and mutant CLPP (c.628G > A) in SVOG cells. **(G)** In KGN cells, the growth and viability of cells were detected and quantified using CCK8. Wild-type CLPP (WT) and mutant CLPP (c.628G > A) were compared with the control group (vector), respectively. **(H)** In SVOG cells, the growth and viability of cells were detected and quantified using CCK8. Wild-type CLPP (WT) and mutant CLPP (c.628G > A) were compared with the control group (vector), respectively.

The pathological basis of POI is a decrease in the number of follicles. The cellular localization and expression dynamics of Clpp in the ovaries of neonatal (Figure 2A) and sexually mature mice (Figure 2B) were detected by immunofluorescence to investigate the role of Clpp in follicular development. It showed that Clpp was mainly localized in the cytoplasm of ovarian granulosa cells and oocytes, and was relatively highly expressed in follicular granulosa cells.

To determine whether the mutation of CLPP at the A210 locus affected the growth of ovarian granulosa cells, we transfected empty vector, wild-type CLPP and mutant CLPP (c.628G > A) into KGN and SVOG cells and assayed the viability of the cells using CCK8 (Figures 2C–F). The results indicated that wild-type CLPP had no effect on the growth of granulosa cells comparing the empty vector. Importantly, this variant remarkably reduced the growth and viability of KGN and SVOG cells (Figures 2G, H).

### 2.3 CLPP regulated the folding of COX5A

**FIGURE 3:**
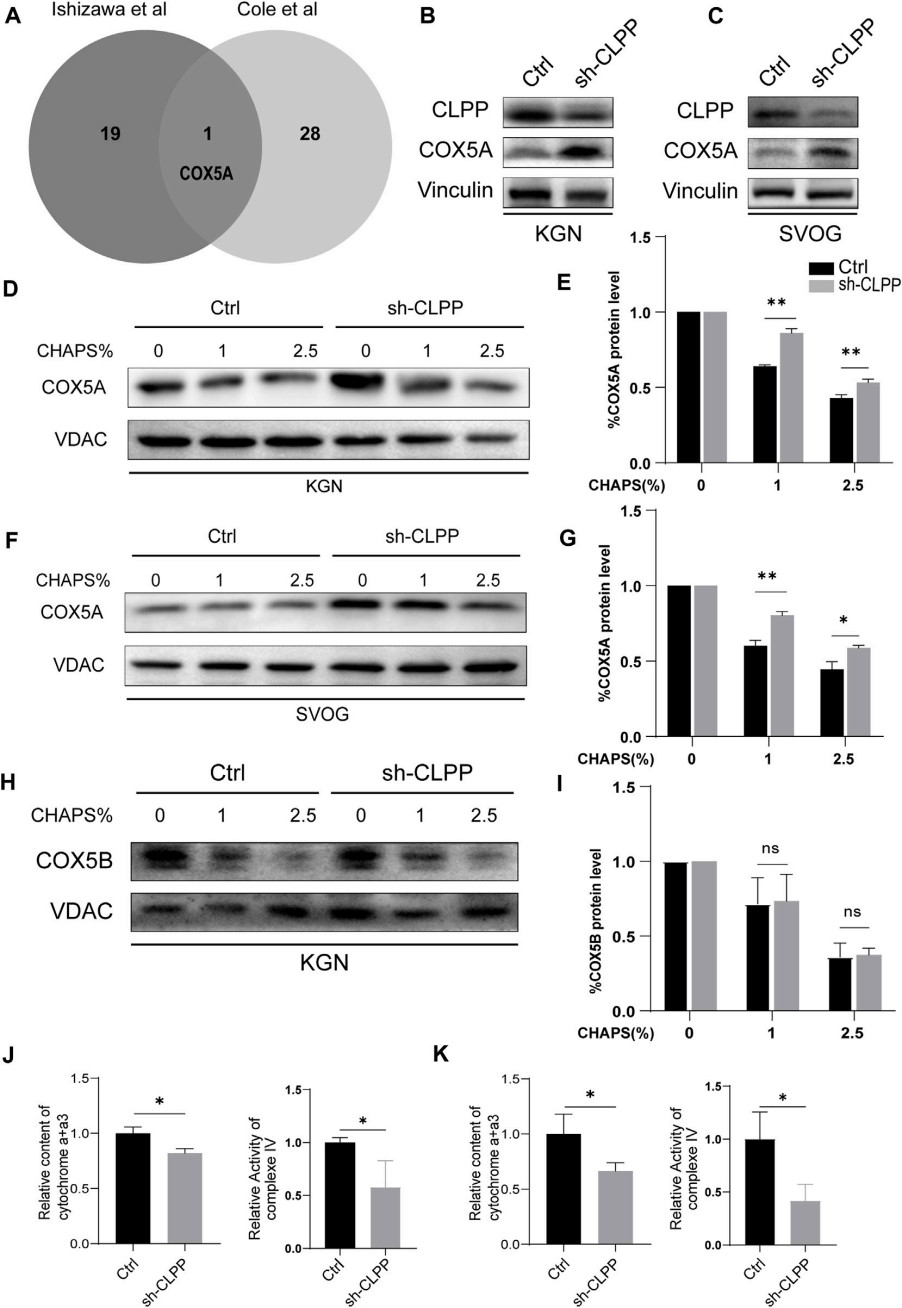
CLPP regulated the folding of COX5A **(A)** Wayne plot showed proteomics data from two published studies investigating CLPP substrates and taking intersections. **(B,C)** shRNA against CLPP (sh-CLPP) and the negative control shRNA (sh-NC) was transfected into KGN **(B)** and SVOG **(C)** cells, respectively, and the knockdown efficiency and COX5A content were analyzed using Western blot. **(D–I)** KGN **(D,E,H,I)** and SVOG **(F,G)** cells were mixed with the indicated increasing concentrations of CHAPS, and the insoluble substance of COX5A or COX5B was analyzed by Western blotting. The density quantification of the bands in COX5A or COX5B was normalized to those in its VDAC, and then the groups with different concentrations of CHAPS (1% and 2.5%) were compared with the group without CHAPS (0) respectively to obtain the ratio of relative density quantification. **(J,K)** The content of cytochrome a+a3 and the activity of complex IV were analyzed in KGN **(J)** and SVOG **(K)** cells.

CLPP acts as a mitochondrial protein controller by degrading unfolded or misfolded proteins in the mitochondria. We analyzed mass spectrometry data from two studies to explore whether the knockdown of CLPP caused the accumulation of unfolded or misfolded proteins (Cole et al., 2015; Ishizawa et al., 2019), both of which were analyzed by BioID combined with mass spectrometry in 293T cells for interacting proteins or substrates of CLPP, and the interactions of these were taken. COX5A was the only intersection of these two datasets (Figure 3A), which is one of the subunits of the respiratory chain complex IV and involved in the maintenance and regulation of mitochondrial function. Previous studies have found that abnormalities in COX5A are associated with growth retardation, abnormal lactate metabolism and aging-related diseases such as Parkinson’s disease and Alzheimer’s disease (Liang et al., 2022; O'Hanlon et al., 2022; Torraco et al., 2022). It is known that POI is also a disease associated with reproductive aging (Ke et al., 2023), however, the relationship between COX5A and POI remains unclear. Therefore, we are interested in exploring the associations and mechanisms of COX5A, a potential substrate of CLPP, in the pathogenesis of POI.

Firstly, we selected human ovarian granulosa cell lines KGN and SVOG as cell models to construct CLPP stable knockdown cell lines to exclude possible off-target effects of siRNA to further explore the functions played by CLPP in ovarian granulosa cells. The results indicated that the silencing of CLPP led to elevated expression of COX5A (Figures 3B, C; Supplementary Figure S1). Next, we examined the folding state of COX5A in the absence of CLPP. We found that the knockdown of CLPP induced the accumulation of misfolded or aggregated COX5A at different CHAPS concentrations under the same conditions (Figures 3D–G). In addition, we assayed the solubility of COX5B, as another possible substrate for CLPP, which is also a component of the respiratory chain complex IV (Lee et al., 2021; Mabanglo et al., 2022). In contrast to COX5A, depletion of CLPP did not affect the solubility of COX5B (Figures 3H, I). These data suggested that CLPP selectively regulates the COX5A protein quality by degrading the unfolded COX5A protein. Finally, the content and activity of mitochondrial respiratory chain complex IV were also tested. It was shown that the silence CLPP resulted in a decrease in the content and activity of mitochondrial respiratory chain complex IV (Figures 3J, K), which might be the evidence of COX5A abnormalities affecting the assembly of complex IV.

### 2.4 Inhibition of CLPP impaired mitochondrial function

**FIGURE 4:**
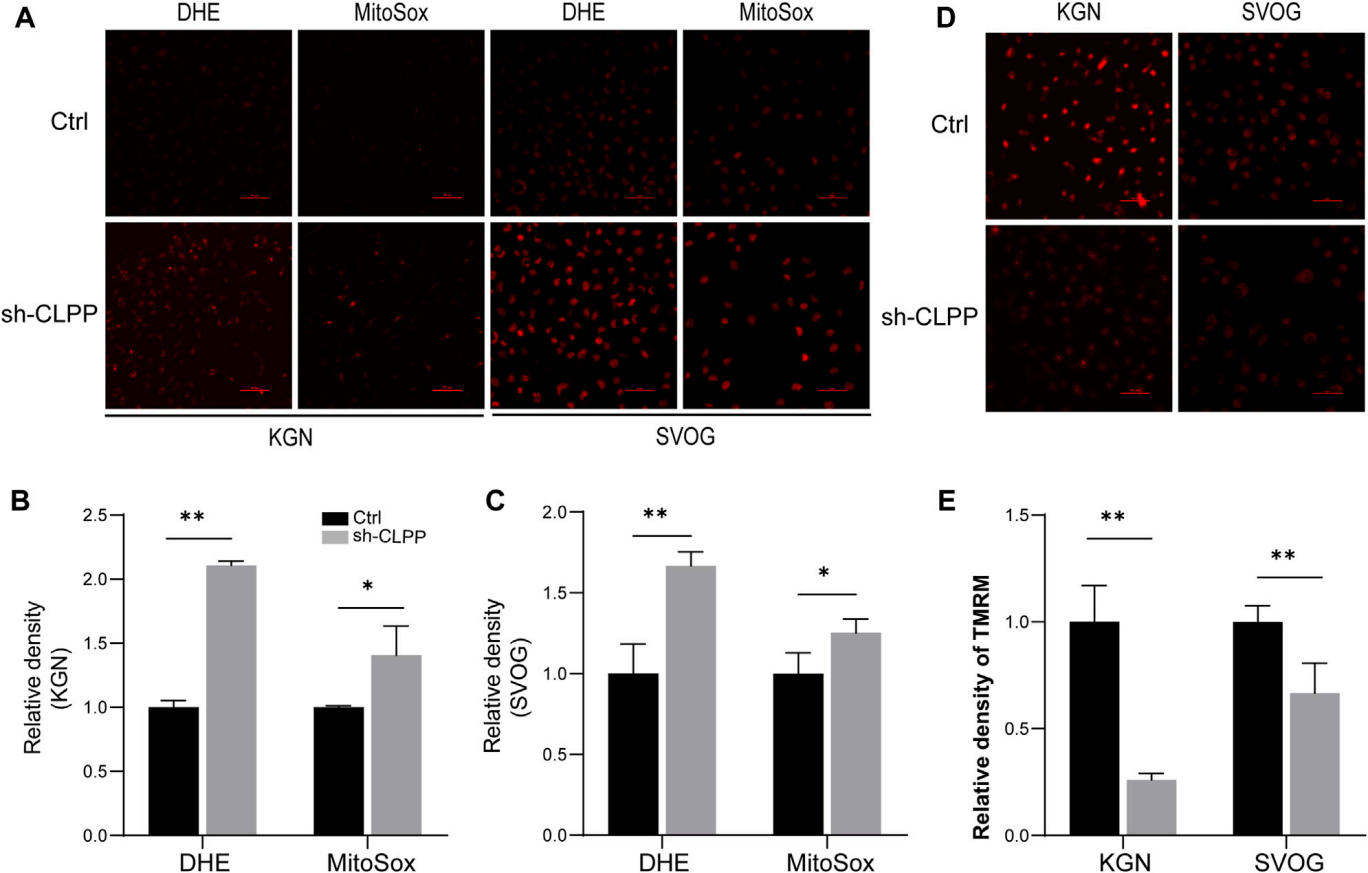
Inhibition of CLPP impaired mitochondrial function **(A–C)** The levels of total ROS and mitochondrial ROS were measured using DHE and MitoSox, respectively. Typical fluorescence images and average fluorescence intensity levels were obtained in KGN **(A,B)** and SVOG cells **(A,C)** for quantitative analysis. **(D,E)** The level of mitochondrial membrane potential was measured using TMRM. Typical fluorescence images and average fluorescence intensity levels were obtained in KGN and SVOG cells for quantitative analysis.

Based on the aforementioned results, we next investigated the downstream consequences of CLPP targeting in cells with defective mitochondrial respiration. The silencing of CLPP resulted in increased production of total cellular superoxide in ovarian granulosa cells. This response was also associated with increased generation of mitochondria-specific reactive oxygen species (ROS) compared with the controls (Figures 4A–C). The mitochondrial membrane potential (ΔΨ) was detected using TMRM staining and detected by flow cytometry. The results showed that the decrease in CLPP protein levels led to a decrease in mitochondrial membrane potential in KGN and SVOG cells compared with the control group (Figures 4D, E).

### 2.5 Gene expression showed changes in granulosa cells

**FIGURE 5:**
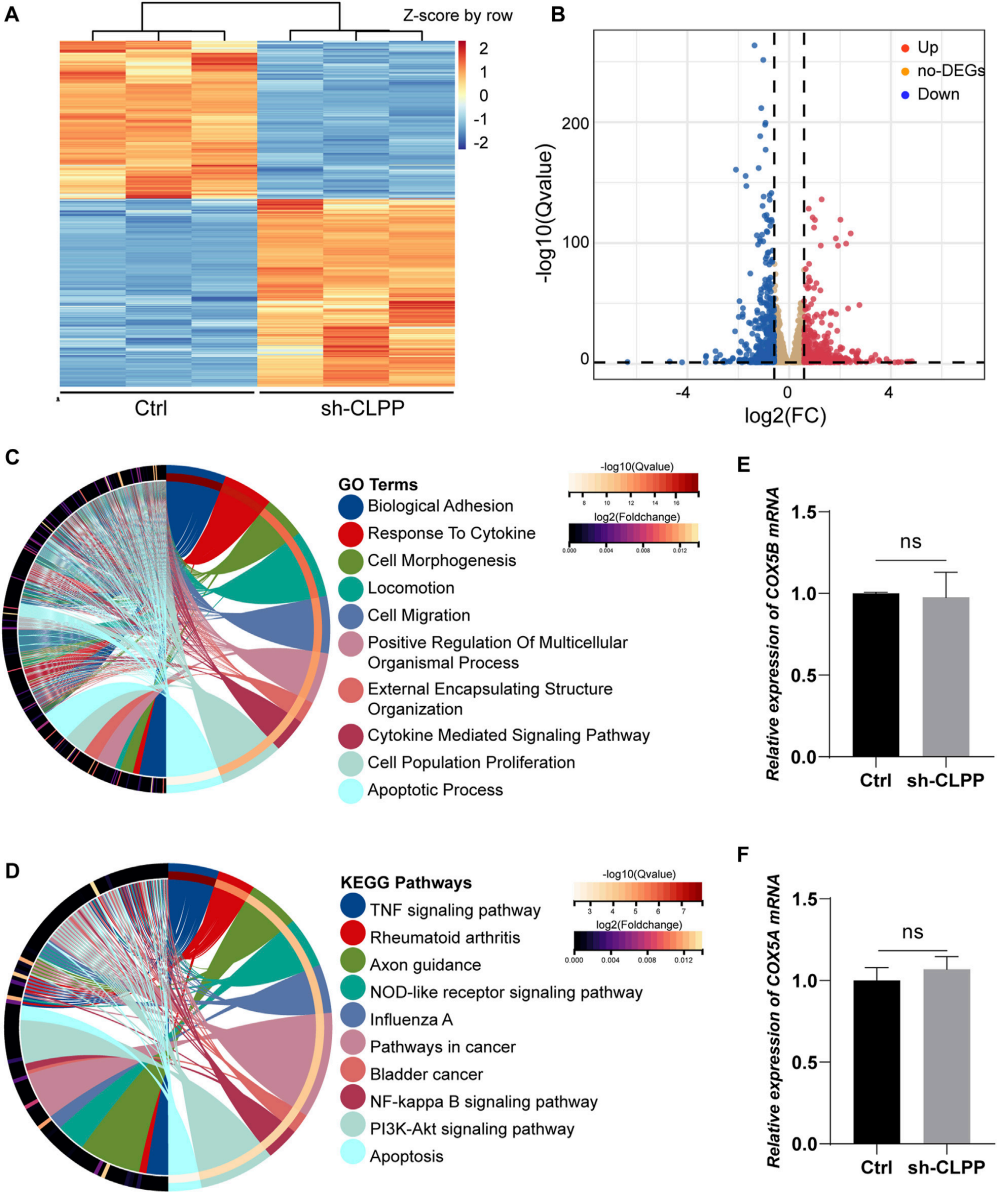
Gene expression showed changes in granulosa cells **(A)** Heatmap illustration showing differentially expressed genes after knockdown of CLPP in KGN cells. The color spectrum from red to blue indicated the normalized level of gene expression from high to low. **(B)** Volcano plots for RNAseq showing DEGs of sh-CLPP compared to Ctrl. Red spots represented Fold Change (FC) > 1.5, blue spots represented FC > 1.5, yellow spots represented FC < 1.5. **(C,D)** GO **(C)** and KEGG **(D)** enrichment analysis of differentially expressed genes (FC > 1.5, Q value ≤ 0.05). **(E,F)** The mRNA levels of COX5A and COX5B were detected using RT-PCR with β-actin as an internal reference.

We performed RNAseq analysis to compare the gene expression in KGN cells after CLPP was silenced. A total of 4,692 genes were significantly differentially expressed after reducing CLPP expression in KGN cells compared with controls (*Q* value ≤ 0.05). Among these genes, 421 were upregulated and 502 were downregulated (FC > 1.5) (Figures 5A, B). GO analysis of significantly differentially expressed genes showed significant changes in genes regulating cell biology adhesion, cytokine response, cell migration, and apoptosis (Figure 5C). Consistent with this finding, KEGG suggested that the apoptosis rate was significantly altered, while the TNF signaling pathway, NF-kappa B signaling pathway, and PI3K–Akt signaling pathway were all affected in KGN cells after CLPP silencing (Figure 5D).

Subsequently, the effect of inhibiting CLPP on the mRNA levels of the components of respiratory chain complex IV was also analyzed. The results showed that none of them expressed differentially (FC > 1.5, *Q* value ≤ *0.05*) (Supplementary Table S2). Additionally, the mRNA levels of COX5A and COX5B were not altered compared to the control group (Figures 5E, F). Concludingly, CLPP had no effect on COX5A mRNA expression.

### 2.6 Knockdown of CLPP induced ovarian granulosa cell apoptosis through a mitochondria-related apoptotic pathway

**FIGURE 6:**
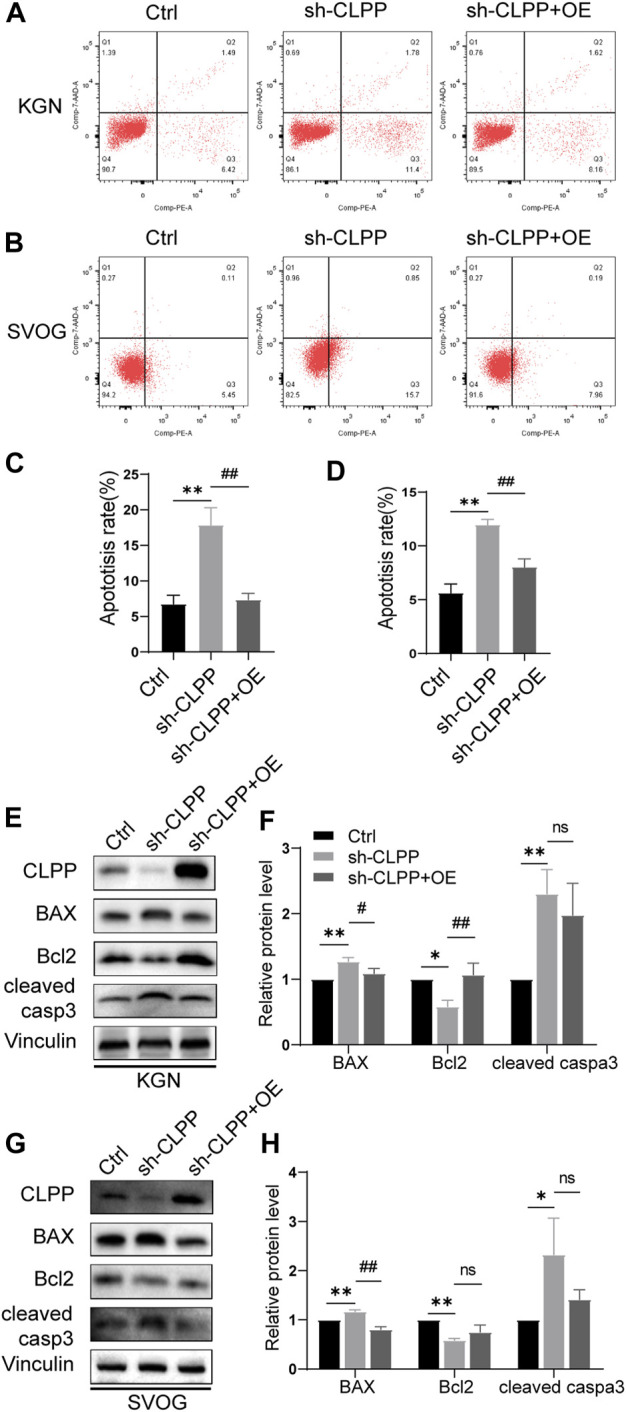
Knockdown of CLPP induced ovarian granulosa cell apoptosis through the intrinsic apoptotic pathways. **(A,B)** Apoptosis signals were detected in KGN **(A)** and SVOG **(B)** cells using Annexin V-PE/7-AAD staining. **(C,D)** Percentages of apoptotic cells were measured by flow cytometry in KGN **(C)** and SVOG **(D)** cells. **(E–H)** KGN cells transfected with CLPP-directed shRNA were re-expressed with CLPP by adenovirus and analyzed using Western blot. BAX, Bcl-2, and cleaved caspase3 protein expression levels in the KGN cells were observed and quantified **(E,F)**. Similar results were observed in SVOG cells **(G,H)**. The asterisk indicates that sh-CLPP is compared with Ctrl. The tic mark indicates that sh-CLPP + OE is compared with sh-CLPP.

The flow cytometry analysis was performed using Annexin/PE and 7-AAD staining to investigate whether CLPP deletion could alter the apoptosis rate in KGN and SVOG cells. The results showed that the silencing of CLPP significantly increased the degree of apoptosis. Next, we re-expressed wild-type CLPP with adenovirus in CLPP-silenced KGN and SVOG cells; the re-expression of CLPP rescued the apoptosis of cells (Figures 6A–D).

In addition, we further assessed the effect of CLPP on apoptosis at the protein level. Specifically, the effect of CLPP on the expression of Bcl-2, BAX, and cleaved caspase 3 proteins was investigated. The results showed that the silencing of CLPP downregulated Bcl-2 expression and upregulated BAX expression in KGN and SVOG cells. The knockdown of CLPP significantly increased the level of cleaved caspase 3 in KGN and SVOG cells compared with that in the control group. The re-expression of CLPP downregulated the protein level of BAX compared with that in the knockdown group (Figures 6E–H; Supplementary Figure S2). The results suggested that the silencing of CLPP induced apoptosis in KGN and SVOG cells through the upregulation of BAX and the activation of caspase3.

## 3 Discussion

CLPP is located on chromosome 19p13.3 and is essentially a component of the ATP-dependent protein hydrolase complex in the mitochondria. The barrel-shaped chamber formed by multiple copies of this protein interacting with each other is known as the CLPP complex, where abnormal proteins are disaggregated into small fragments (Kang et al., 2005). Mitochondria is known as a power generator in eukaryotic cells, and its basic function is to produce ATP through an oxidative phosphorylation (OXPHOS) process (Pfanner et al., 2019). The aberrant expression of oxidative phosphorylation complexes due to any reason can induce increased production of ROS, which significantly affects mitochondrial function (Shpilka and Haynes, 2018). The mitochondrial unfolded protein response (UPR^mt^) is a transcriptional response activated by several forms of mitochondrial dysfunction and regulated by mitochondrial-to-nuclear communication. This fulfills the function of correctly folding proteins and facilitating the removal of misfolded proteins, thus ensuring the accuracy and integrity of the mitochondrial proteome (Shpilka and Haynes, 2018). In eukaryotic cells, CLPP is an essential component of the mitochondrial unfolded protein response signaling pathway, mainly in degrading damaged proteins and maintaining protein homeostasis in mitochondria. Proteomic studies provide a map of mitochondrial CLPP substrates. CLPP has a broad substrate pool and is involved in several biological processes in mitochondria, such as oxidative phosphorylation, translation, Krebs cycle, fatty acid metabolism, and amino acid metabolism (Mabanglo et al., 2022). In the prostate cancer cells, the silencing of CLPP caused a decrease in the activity of complex II due to the accumulation of SDHB, but had no significant effect on other complexes (Ishizawa et al., 2019). In addition, the protein levels of complexes I and IV were reduced in the testes of knockout mice (*Clpp* ^−/−^), while a similar level of expression was not found in the brain and heart (Gispert et al., 2013). These findings suggested cellular and tissue heterogeneity in regulating CLPP on substrate proteins.

Cytochrome c oxidase (COX), also known as complex IV, is thought to be the central regulatory site of OXPHOS (Arnold, 2012). It primarily catalyzes the transfer of electrons from cytochrome c to oxygen and is a focal site for regulating oxidative phosphorylation and ATP and ROS production (Arnold, 2012). COX deficiency adversely affects energy metabolism, leading to serious pathological conditions such as Leigh syndrome, cardiomyopathy, and lactic acidemia (Smeitink et al., 2001). COX5A is one of the major components of respiratory chain complex IV and is also involved in the maintenance of mitochondrial function. COX5A is engaged in the regulation of cell death. The downregulation of COX5A expression increases the apoptosis rate at the onset of myocardial infarction (Jung et al., 2021). In contrast, the overexpression of COX5 protects cortical neuronal activity during an ischemic injury in the brain (Jiang et al., 2020). In addition, COX5A is also associated with aging-related diseases. The overexpression of COX5A is involved in ameliorating memory dysfunction associated with brain aging by affecting the BDNF/ERK1/2 signaling pathway (Xiyang et al., 2020). So far, the association between CLPP and COX5A has not been studied. Our study found that COX5A was an important substrate for CLPP and affected ovarian granulosa cell survival. The inhibition of CLPP leads to the accumulation of impaired COX5A, which affects the function of complex IV and causes oxidative stress and mitochondrial damage. Stabilizing mitochondrial proteins regulated by CLPP is crucial for maintaining the activity of ovarian granulosa cells. We hypothesized that the imbalance of protein homeostasis in mitochondria, including COX5A, might also contribute to the abnormal ovarian function triggered by CLPP abnormalities.

The mutations in CLPP lead to the development of Perrault syndrome type 3, in which the main clinical manifestations are ovarian insufficiency and varying degrees of hearing loss (Dursun et al., 2016). Other non-specific clinical manifestations include cerebellar ataxia, mental retardation, epilepsy, somatic wasting, peripheral neuropathy, and muscle atrophy (Jenkinson et al., 2013). The present case reported a new CLPP variant with clinical manifestations of reduced ovarian function and infertility without hearing impairment or other clinical manifestations. So far, her clinical presentation does not seem to be consistent with the diagnosis of the syndrome. Subsequent follow-up of her hearing status is still needed in the future. To date, 13 different variants of CLPP have been associated with Perrault syndrome, which is genetically heterogeneous (Faridi et al., 2022). The clinical presentation varies significantly, and the different variants may be strongly related to disease severity observed in patients. A pure mutation in CLPP was found in a Pakistani consanguineous family (Jenkinson et al., 2013). The youngest daughter presented with striated ovaries and hypergonadotropic hypogonadism. Her sister presented with erratic menstrual cycles and secondary amenorrhea. The oldest female patient was diagnosed with POI at 22 years of age with early menopause, but notably, two sons were born prior to her diagnosis. Thus, the screening for the genetic variants of CLPP may be clinically relevant in patients with unexplained gonadal dysfunction or hearing impairment. In patients in which hearing impairment is the main manifestation, it is also necessary to perform tests of reproductive function. Moreover, in patients with early diagnosis, early fertility preservation may be an option worth considering, provided the ovaries are present.

A mouse knockout of *Clpp* has been generated. The number of mature oocytes and two-cell embryos in mice is lower, with no blastocysts. These deficiencies in oocyte and embryo development are related to impaired mitochondrial function and dynamics (Wang et al., 2018). Meanwhile, the apoptotic signals in the follicular granulosa cell layer are significantly increased and show accelerated depletion of follicular reserves and hearing loss, consistent with the symptoms of Perrault syndrome (Gispert et al., 2013; Wang et al., 2018). Lower proliferation or excessive apoptosis of ovarian granulosa cells may result in a decrease in the number of cell connections or damage to cell connections, which leads to a lack of nutrients and factors necessary for oocyte growth, causing follicular atresia (Nesvizhskii, 2007). In our study, CLPP mutation decreased the growth and viability of KGN cells. A similar result was observed in SVOG cells. Simultaneously, the inhibition of CLPP elicited elevated levels of ROS and decreased mitochondrial membrane potential. The transcriptomic sequencing revealed that the change in CLPP resulted in alterations in the apoptotic pathway, clarifying the role of CLPP in ovarian granulosa cells. The silencing of CLPP was confirmed by flow cytometry to cause increased apoptosis. Additional assays using Western blot showed that the knockdown of CLPP caused the upregulation of Bax and cleaved caspase3, while Bcl-2 expression was downregulated, implying the activation of the intrinsic apoptotic pathways. It suggested that the knockdown of CLPP in granulosa cells might exert an adverse effect on follicular development. Ecem et al. created a *Clpp* ^flox^/*Cyp19a1*-Cre mouse model to specifically delete *Clpp* in the granulosa/cumulus cells. However, no impairment in fertility, follicle development, or the number of follicles in each stage was found in mature female mice. It suggested that the expression of CLPP in follicular somatic cells was not required for fertility (Esencan et al., 2020). Lv et al. (2019) investigated the role of Hippo signaling pathway in granulosa cells during follicle development by constructing a conditional knockout mouse model of ovarian granulosa cells. The results revealed that *Foxl2* promoter–driven knockdown of Yap1 in ovarian granulosa cells resulted in increased follicular apoptosis, decreased number of corpus luteum, reduced ovary size, and low fertility. However, *Cyp19a1* promoter–driven Yap1 knockdown did not affect ovarian morphology and fertility. Cyp19a1 is mainly expressed in the differentiated granulosa cells. Therefore, *Cyp19a1*-CRE mice may be better used in examining the function of specific gene loss in well-differentiated granulosa cells and luteal cells (Lv et al., 2019). Foxl2 belongs to the family of winged-helix/forkhead transcription factors, and it is expressed in ovarian follicle somatic cell lineages, including the primordial granulosa cells in primordial follicles and the granulosa cells in growing follicles (Zhang and Liu, 2015). Therefore, *Foxl2*-CRE mice may be used to explore the impact of CLPP gene deletion in granulosa/cumulus cells on follicular development.

In conclusion, we identified a new variant of CLPP associated with POI. Also, we confirmed that CLPP deficiency in ovarian granulosa cells decreased the function of oxidative respiratory chain IV by affecting COX5A, leading to the accumulation of ROS. Additionally, the intrinsic apoptotic pathways can be affected by CLPP to regulate the apoptosis of ovarian granulosa cells. We confirmed the effect of CLPP on ovarian granulosa cells at the cellular level. Also, we hypothesized that this might be a potential mechanism by which CLPP abnormalities caused accelerated ovarian follicular atresia. Further studies with primary ovarian granulosa cells and ovarian granulosa cell conditional knockout models (FOXL2-CRE) are required to validate our results.

## 4 Materials and methods

### 4.1 Whole-exome sequencing and analyses

To identify the causative gene in this patient, genomic DNA was extracted from the peripheral blood of the patient, her brother and her parents with their permission, and then analyzed by whole-exome sequencing by Kaiumph Medical Diagnostics (Beijing, China). The pathogenicity of the CLPP mutant locus was predicted using bioinformatics tools and the mutant loci were validated using Sanger sequencing. Functional pathogenicity of the variants was predicted by online bioinformatics tools such as Polyphen-2 (Adzhubei et al., 2010), SIFT (Kumar et al., 2009), and Mutation Taster (Schwarz et al., 2014). This study was also approved by the Ethics Committee of the First Affiliated Hospital of Nanjing Medical University.

### 4.2 Animals

Adult C57BL/6 mice were purchased from Weitong Lihua Limited Company (Beijing, China). Female C57BL/6 mice and bred to male mice of the same strain. They had free access to water and food and were housed under appropriate light (12 h light, 12 h dark) and temperature (24°C–26°C) conditions. Detection of vaginal plugs was considered to be 0.5 days post coitus (dpc). The first 12 h after birth were considered as 0 days *postpartum* (dpp). All animal experiments were approved by the Institutional Animal Care and Use Committee (IACUC) of Nanjing Medical University, and the experimental methods were performed according to the approved protocols.

### 4.3 Immunofluorescence

The mice were sacrificed at the indicated times and ovaries were fixed in 4% paraformaldehyde for 24 h, followed by histological processing according to standard procedures. Briefly, ovaries are typically paraffin-embedded and histologically sectioned, followed by antigen repair, blocking, and incubation of primary antibodies at 4°C overnight and secondary antibodies at room temperature for 1 h the next day. Hoechst was used for nuclear staining. Immunofluorescence signals were obtained using confocal microscopy.

### 4.4 Cell culture

KGN and SVOG cells were obtained from the State Key Laboratory of Reproductive Medicine of Nanjing Medical University. Cells were cultured in DMEM/F-12 medium (Zhongqiao Xinzhou Biotechnology Co., Shanghai, China) supplemented with 10% heat-inactivated fetal bovine serum (FBS) (Gibco) and 100 U/mL penicillin/streptomycin (Gibco) at 37°C under a humidified atmosphere of 5% CO_2_.

### 4.5 Transfection

Short hairpin RNA against CLPP (sh-CLPP) or CLPP overexpressing adenovirus and respective negative controls were synthesized by Vigene Biology (Shandong, China). The RNA sequence of sh-CLPP was listed in the Supplementary Table S1. In addition, the pcDNA3.1 vector with the full-length cDNA sequence of wild-type CLPP and the point mutant CLPP (c.628G > A) and the empty pcDNA3.1 vector were also provided by Vigene.

Lentiviral sh-CLPP or its negative control was transfected into cells according to the manufacturer’s protocol, then 2 μg/mL puromycin was added to kill untransfected cells after 48 h. When the density of cells reached 50%–60%, adenovirus was added to them to overexpress wild-type CLPP. When cells reached 70%–80% confluency, plasmids were transfected using Lipo8000™ Transfection Reagent (Beyotime).

### 4.6 Cell viability

Cell viability was analyzed by Cell Counting Kit-8 (CCK-8, APExBIO, United States). Cells (5,000 cells/well) were seeded into 96-well plates for 12 h, followed by vector and mutant plasmid transfection. The transfected cells were incubated for 24 h, 48 h, and 72 h, followed by incubation with 10 μL CCK-8 reagent for 1 h. The absorbance at 450 nm was measured using a microplate reader (MuLTiSKAN, Thermo, United States).

### 4.7 Western blot

The proteins were extracted from cells in each group using RIPA buffer, and 30 μg of total lysate obtained from KGN cells was loaded into sodium dodecyl sulfate polyacrylamide gel electrophoresis (SDS-PAGE). Antibodies used in this study were listed in Supplementary Table S1.

### 4.8 Mitochondrial protein folding

Mitochondrial protein folding experiments were performed as described previously (Chae et al., 2013; Lee et al., 2021; Seo et al., 2016). Mitochondrial fractions were extracted using the Mitochondrial Isolation Kit (Beyotime) according to the manufacturer’s recommendations. The mitochondrial fraction was suspended in an equal volume of mitochondrial storage solution supplemented with increasing concentrations of CHAPS (0%, 1% or 2.5%). Samples were incubated by lysis on ice for 30 min, and detergent-insoluble protein aggregates were recovered by centrifugation (20,000 g) for 20 min. The particulate proteins were separated by SDS-PAGE.

### 4.9 Detection of respiratory chain complex IV activity

The activity of complex IV in the mitochondrial electron respiratory chain was determined using the Mitochondrial Respiratory Chain Complex IV Activity Assay Kit (Abbkine, Wuhan, China) according to the manufacturer’s instructions. Briefly, Five million cells were collected for mitochondrial extraction on ice. The resulting mitochondrial precipitate is resuspended in extraction buffer and resuspended. The microplate reader (MuLTiSKAN, Thermo, United States) was pre-warmed at 37°C for 30 min and then the activity of complex IV in the corresponding reaction buffer was measured at 550 nm.

### 4.10 Measurement of cytochrome a+a3 content

The content of cytochrome a+a3 was estimated as previously described (Dejean et al., 2000). Briefly, cells were harvested and washed twice with distilled water to obtain 2 mL of cell suspension with approximately 50 optical density (OD) units at 600 nm. A blank control assay was then performed. 1 ul of 70% (w/v) H_2_O_2_ was added to 500 µL of distilled water and the absorbance was measured at 603 nm with a spectrophotometer (blank oxidized state). An appropriate amount of sodium dithionite was added to another 500 µL of distilled water and similar measurements were also performed (blank reduced state). Moreover, the absorbance values of the oxidized and reduced states of the 500 µL of cell suspension were measured respectively using the same method. Relative content of cytochrome a+a3 = [OD (reduced state)-OD (blank reduced state)]/[OD (oxidized state)-OD (blank oxidized state)].

### 4.11 Assessment of ROS content and mitochondrial membrane potential

Briefly, cells were incubated with 10 μM DHE (KeyGEN Biotech, China) or 5 μM MitoSOX RED (Invitrogen, United States) or 25 nM TMRM (Invitrogen, United States) for 30 min and washed 3 times with PBS. Fluorescence signals were detected by confocal microscopy. Data were analyzed using ImageJ software.

### 4.12 RNA sequence and analysis

The total RNA extraction, RNA sequence and bioinformatics analysis were all done by BGI (BGI, Wuhan, China).

Total RNA was extracted from cells using Trizol (Invitrogen, United States) according to the manual instructions. It was characterized and quantified using a Nano Drop and Agilent 2,100 Bioanalyzer (Thermo Fisher Scientific, MA, United States). Oligo (dT)-attached magnetic beads were used to purify mRNA, followed by reverse transcription and PCR amplification, and the product was purified by Ampure XP beads and then dissolved in EB solution. The products were verified on an Agilent Technologies 2,100 Bioanalyzer for quality control. The double-stranded PCR products from the previous step were denatured by heating and cyclized by grafted oligosaccharide sequences to obtain the final library. Single-stranded circular DNA (ssCir DNA) was formatted as the final library. The final library was amplified with phi29 into DNA nanoballs (DNBs), where one molecule has more than 300 copies, and the DNBs were loaded into patterned nanoarrays to generate single-ended 50-base reads on the BGIseq500 platform (BGI-Wuhan, China).

The sequencing data was filtered with SOAPnuke (v1.5.2) by 1) Removing reads containing sequencing adapter; 2) Removing reads whose low-quality base ratio (base quality less than or equal to 5) is more than 20%; 3) Removing reads whose unknown base (‘N’ base) ratio is more than 5%, afterwards clean reads were obtained and stored in FASTQ format (Li et al., 2008). The clean reads were mapped to the reference genome using HISAT2 (v2.0.4). Bowtie2 (v2.2.5) was applied to align the clean reads to the reference coding gene set, then expression level of gene was calculated by RSEM (v1.2.12) (Kim et al., 2015; Li and Dewey, 2011). Essentially, differential expression analysis was performed using DESeq2 (v1.4.5) with a *Q* value ≤ 0.05. Volcano and heat maps were drawn according to the gene expression of different samples using the OmicStudio tools at https://www.omicstudio.cn/tool. To gain insight into the phenotypic changes of differentially expressed genes, GO and KEGG enrichment analysis of the annotated differentially expressed genes was performed using Sangerbox (Shen et al., 2022). All sequencing data are available through the NCBI Sequence Read Archive under the accession number PRJNA883425.

### 4.13 RNA extraction and RT-PCR assay

Cells were obtained at the appropriate time and total RNA was extracted using the RNA-Quick extraction kit (ES Science, Shanghai, China). Next, cDNA was synthesized using TaKaRa Reverse Transcription Kit (Takara, Dalian, China). Finally, the StepOne Plus Real-Time PCR system and StepOne Plus software were applied for DNA amplification and data analysis. Relative expression levels of mRNA were calculated using a relative quantification method (2^−ΔΔCt^), and β-actin was used as an internal reference. The sequences of the primers used in this study are shown in Supplementary Table S1.

### 4.14 Evaluation of cell apoptosis

Apoptosis was detected by flow cytometry. Cells were seeded into 6-well plates. Cells were digested with trypsin and resuspended in binding buffer to prepare single cell suspensions and stained using Annexin V-PE/7-AAD reaction reagent (Vazyme, A213, Nanjing, China) for 5 min at room temperature in the dark. The stained cells were then analyzed with BD Accuri C6 Plus (BD Biosciences). The results were analyzed with FlowJo v10.5 software.

### 4.15 Statistical analysis

All graphs and statistical analyses were performed using Graph Pad Prism version 7 software. Data were analyzed using Student *t*-test, one-way ANOVA test. Unless otherwise stated, data represent three independent experiments. Differences were considered statistically significant for *p* < 0.05. An asterisk (*) or a tic mark (#) indicates a significant difference at *p* < 0.05; double asterisks (**) or tic marks (##) indicate a significant difference at *p* < 0.01.

## Data Availability

The datasets presented in this study can be found in online repositories. The names of the repository/repositories and accession number(s) can be found below: https://www.ncbi.nlm.nih.gov/, PRJNA883425.
