# Supplementary material for: CLPP inhibition triggers apoptosis in human ovarian granulosa cells via COX5A abnormality–Mediated mitochondrial dysfunction
Source: Front Genet. 2023 Mar 17;14:1141167. doi: 10.3389/fgene.2023.1141167 (PMC10065195; doi:10.3389/fgene.2023.1141167)
Supplement: Supplementary file 2 [file Presentation1.PDF]

Supplementary Material

CLPP inhibition triggers apoptosis in human ovarian granulosa cells via COX5A abnormality–mediated mitochondrial dysfunction

Xiong Yuan[1,2], Wenjie Ma[1,2], Shuping Chen[1,2], Huiyuan Wang[1,2], Chenyi Zhong[1,2], Li Gao[1,2], Yugui Cui[1,2], Danhua Pu[1,2], Rongrong Tan*[1,2] and Jie Wu*[1,2]

[1]Department of Obstetrics and Gynecology, The First Affiliated Hospital of Nanjing Medical University, Nanjing, China; [2]State Key Laboratory of Reproductive Medicine, School of Public Health, Nanjing Medical University, China

*** Correspondence:**
Jie Wu* and Rongrong Tan*

wujiemd@126.com; tanrongrong86119@163.com

Supplementary Figures

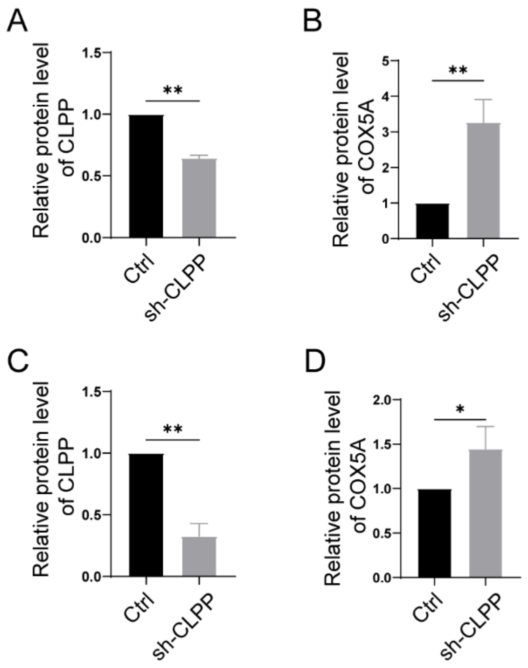

Supplementary Figure 1. CLPP regulated the folding of COX5A

(A-D) Quantitative analysis of the efficiency of CLPP knockdown and the levels of COX5A in KGN (A and B) and SVOG (C and D).

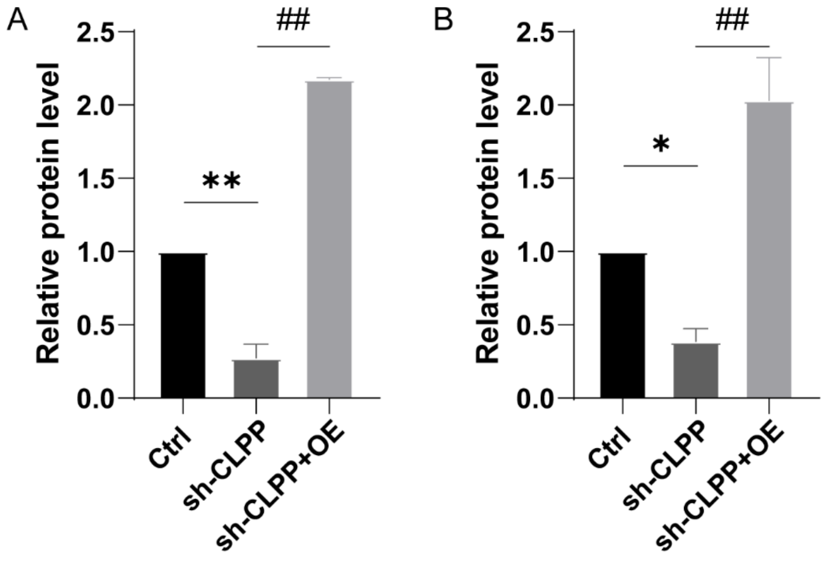

Supplementary Figure 2. Transfection efficiency assays.

(A) KGN cells transfected with CLPP-directed shRNA were re-expressed with CLPP by adenovirus and the levels of CLPP were analyzed using Western blot. (B) Similar results were observed in SVOG cells.
